# The performance of the SEPT9 gene methylation assay and a comparison with other CRC screening tests: A meta-analysis

**DOI:** 10.1038/s41598-017-03321-8

**Published:** 2017-06-08

**Authors:** Lele Song, Jia Jia, Xiumei Peng, Wenhua Xiao, Yuemin Li

**Affiliations:** 1 Department of Radiotherapy, The Chinese PLA 309th Hospital, Beijing, 100091 P.R. China; 2 BioChain (Beijing) Science and Technology, Inc., Beijing, 100176 P.R. China; 3 Department of Graduate, Hebei North University, Zhangjiakou, Hebei P.R. China; 4 Medical School of Chinese PLA and Chinese PLA General Hospital, Beijing, P.R. China; 5 Department of Oncology, First Affiliated Hospital of Chinese PLA General Hospital, Beijing, P.R. China

## Abstract

The SEPT9 gene methylation assay is the first FDA-approved blood assay for colorectal cancer (CRC) screening. Fecal immunochemical test (FIT), FIT-DNA test and CEA assay are also *in vitro* diagnostic (IVD) tests used in CRC screening. This meta-analysis aims to review the SEPT9 assay performance and compare it with other IVD CRC screening tests. By searching the Ovid MEDLINE, EMBASE, CBMdisc and CJFD database, 25 out of 180 studies were identified to report the SEPT9 assay performance. 2613 CRC cases and 6030 controls were included, and sensitivity and specificity were used to evaluate its performance at various algorithms. 1/3 algorithm exhibited the best sensitivity while 2/3 and 1/1 algorithm exhibited the best balance between sensitivity and specificity. The performance of the blood SEPT9 assay is superior to that of the serum protein markers and the FIT test in symptomatic population, while appeared to be less potent than FIT and FIT-DNA tests in asymptomatic population. In conclusion, 1/3 algorithm is recommended for CRC screening, and 2/3 or 1/1 algorithms are suitable for early detection for diagnostic purpose. The SEPT9 assay exhibited better performance in symptomatic population than in asymptomatic population.

## Introduction

Colorectal cancer (CRC) has become the 3^rd^ leading cause of new cancer cases in the world. The prevention of CRC should aim at early detection, which can be achieved by regular screening. Statistics from U.S. Preventive Services Task Force (USPSTF) shows that approximately 60% CRC deaths could be avoided if a regular periodic screening was carried out each year, and the average five-year survival rate could be increased from 46 to 73%^1^. Therefore, effective CRC early screening methods can prolong patients’ lives and reduce mortality. The latest recommendation statement from the USPSTF listed the stool-based gFOBT (guaiac-based fecal occult blood test), FIT (fecal immunochemical test), FIT-DNA (multitargeted stool DNA) tests, the blood-based SEPT9 gene methylation assay and the direct visualization tests (including colonoscopy, CT colonoscopy and sigmoidoscopy) as the current CRC screening strategies^2^.

The blood-based *SEPT9* gene methylation assay aim at detecting the aberrant methylation at the promoter region of the *SEPT9* gene DNA released from CRC cells into the peripheral blood (i.e. the circulating tumor DNA, or ctDNA)^3, 4^. The development of the test is based on a key theory indicating that the detection of the SEPT9 gene aberrant methylation reflects the existence of CRC. The CpG island 3 at the promoter region of the *SEPT9* gene V2 transcript is hypermethylated, and DNA of the gene is released into the peripheral circulating blood from necrotic and apoptotic cancer cells during CRC carcinogenesis^5^. The risk of CRC can be determined by detecting the degree of DNA methylation of the specific promoter region of the *SEPT9* gene in the peripheral blood^6^.

There are 25 independent studies performed so far to investigate the performance of the SEPT9 gene methylation assay in CRC detection, in which most studies were case-control or cohort studies, while only one randomized multi-center screening study and two opportunistic screening studies were performed to investigate its performance in average-risk population and high-risk population, respectively^7, 9–11^. However, the algorithm (1/3 algorithm) used in the screening study was also applied in some other studies, and their performance was therefore comparable to each other. Other algorithms, including 2/3, 1/1 and 1/2, were also used in some studies. There is no consensus on which algorithm is superior in various occasions. Moreover, as FIT and FIT-DNA tests are also CRC screening assays currently used, it would be useful to compare their performance with the SEPT9 assay.

In this systematic review and meta-analysis, we investigated the performance of SEPT9 assay with specific focuses on test sensitivity and specificity at various algorithms. The overall test performance and the stage-dependent sensitivity with different algorithms were compared. We also compared the assay performance with that of the serum protein tumor markers, FIT and FIT-DNA tests. The objective of this study is to identify the best algorithm for various scenarios, and to highlight the pros and cons of the SEPT9 assay in CRC screening.

## Results

### Eligibility of studies and literature characteristics included in the meta-analysis

**Figure 1:**
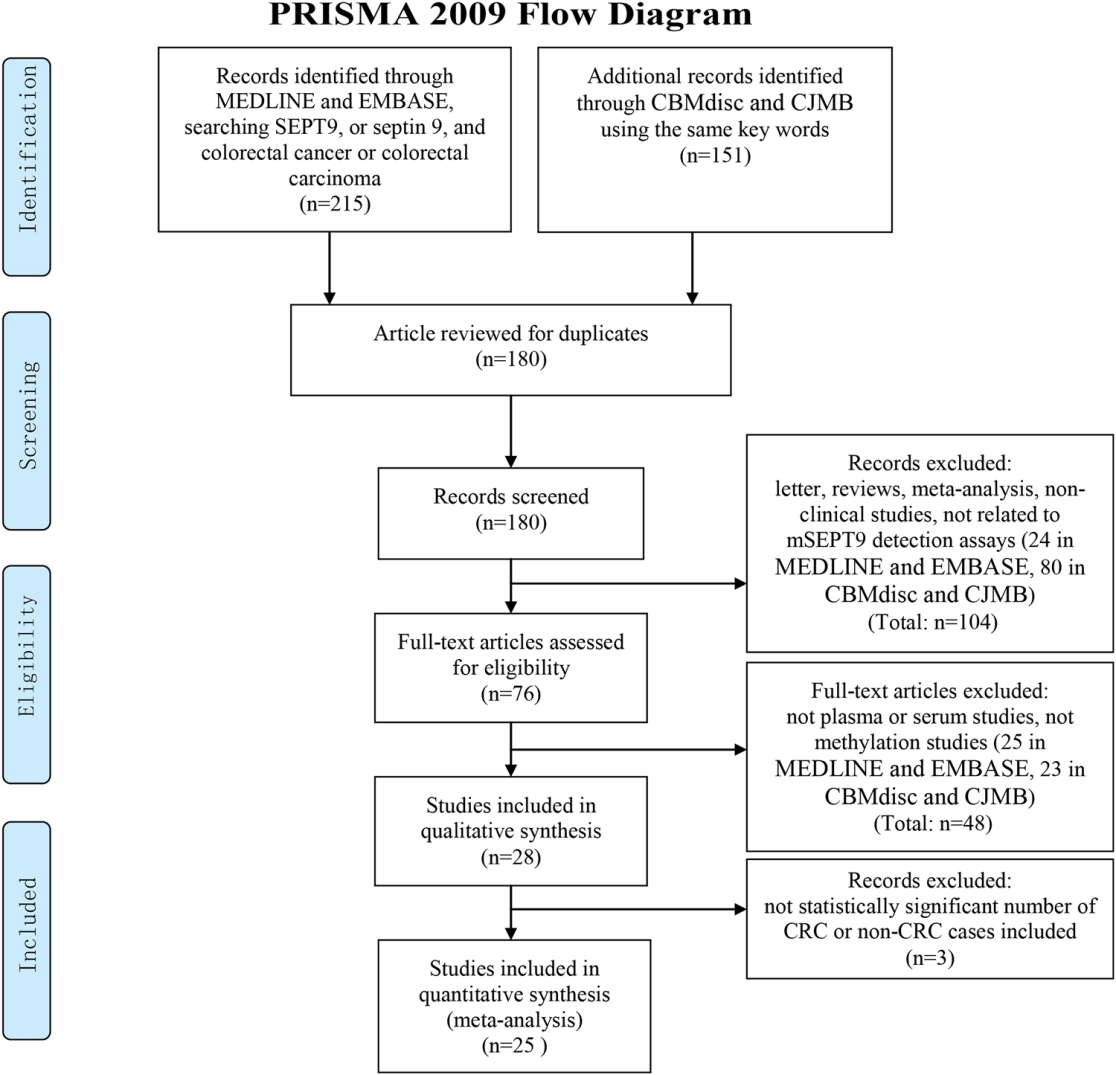
The PRISMA flow diagram for literature selection from relevant studies for this meta-analysis. The design of the diagram and the screening of the literatures were based on the PRISMA statement for reporting systematic reviews and meta-analysis^30, 31^.

Twenty-five studies investigating the performance of the SEPT9 assay at various settings are obtained from the screening through PRISMA flow diagram (Fig. 1) and are listed in Table 1. These studies were accessed using the QUADAS system^8^ and the results were shown in Supplementary Figure 1. Several parameters, including sensitivity, specificity, positive likelihood ratio (PLR), negative likelihood ratio (NLR), odds ratio (OR), algorithm, and kit used in each study, were compared in Table 1. In the 25 reports, 22of them were cohort or case-control studies, while the PRESEPT study is the only one carried out so far in the screening background in average-risk population^7, 9^, and the RESEPT study and a recent report by Song *et al*. performed the opportunistic screening in high-risk population^10, 11^. 3 studies adopted the 1/3 algorithm alone^9, 12, 13^, 3 studies adopted the 1/2 algorithm alone^7, 14, 15^, 6 studies adopted the 1/1 algorithm alone^3, 10, 16–19^, and 7 studies adopted the 2/3 algorithm alone^4, 20–25^, while 6 studies tested both 1/3 and 2/3 algorithm^6, 11, 26–29^.

**Table 1:** Sensitivity, specificity, PLR, NLR and OR of the blood-based SEPT9 gene methylation assay in CRC detection or screening with various algorithm.

Publications	Number of cases	Sensitivity	Specificity	PLR	NLR	OR	Algorithm	Kit used
Grutzmann *et al*., 2008	309 (126 CRC, 183 control)	71.4% (90/126)	89.6% (164/183)	6.93	0.31	22.20	2/3	Research kit
Lofton-Day *et al*.^3^	312 (133 CRC, 179 control)	69% (92/133)	86% (154/179)	4.95	0.36	13.82	1/1	Research kit
deVos *et al*.^26^	514 (187 CRC, 327 control)	73.8% (138/187)	86.2% (282/327)	5.36	0.30	17.65	1/3	Research kit
		56.1% (105/187)	96.6% (316/327)	16.69	0.45	36.78	2/3	
He *et al*., 2010	352 (182 CRC, 170 NED)	74.7% (136/182)	96.5% (164/170)	21.17	0.26	80.81	1/1	Research kit
Weiss and Rosch, 2010	257 (103 CRC, 154 NED)	67.0% (69/103)	87.7% (135/154)	6.10	0.37	16.45	1/2	Epi proColon 1.0
Tanzer *et al*., 2010	67 (33 CRC, 34 control)	82% (27/33)	88% (30/34)	6.95	0.21	33.75	1/3	Epi proColon 1.0
		73% (24/33)	91% (31/34)	8.24	0.30	27.56	2/3	
Warren *et al*., 2011	144 (50 CRC, 94 control)	90.0% (45/50)	88.0% (83/94)	7.69	0.11	67.91	1/3	ARUP Lab LDT assay
		76.0% (38/50)	99.1% (93/94)	71.44	0.24	294.50	2/3	
Ahlquist *et al*., 2012	73 (30 CRC, 43 control)	60.0% (18/30)	79.1% (34/43)	2.87	0.51	5.67	1/3	ARUP Lab LDT assay
Toth *et al*., 2012	184 (92 CRC, 92 control)	95.6% (88/92)	84.8% (78/92)	6.29	0.05	122.57	1/3	Epi proColon 2.0
		79.3% (73/92)	99% (91/92)	73.00	0.21	349.63	2/3	
Wang *et al*., 2012	56 (36 CRC, 20 control)	69.4% (25/36)	90.0% (18/20)	6.94	0.34	20.45	1/1	research kit
Liu *et al*., 2013	57 (37 CRC, 20 control)	54.1% (20/37)	90.0% (18/20)	5.41	0.51	10.59	1/2	research kit
Church *et al*.^7^	1510 (53 CRC, 314 AA, 209 NAA, 934 NED)	48.2% (27/53)	91.5% (1333/1457)	5.67	0.57	10.02	1/2	Epi proColon 1.0
		63.9% (34/53)	88.4% (1288/1457)	5.53	0.41	13.64	1/3	
Potter *et al*., 2014	1544 (44 CRC, 621 AA,435 SP, 444 NED)	68.2% (30/44)	80.0% (1200/1500)	3.41	0.40	8.57	1/3	Epi proColon 2.0
Johnson *et al*.^13^	301 (101 CRC, 200 AA, SP and NED)	73.3% (74/101)	81.5% (163/200)	3.96	0.33	12.07	1/3	Epi proColon 2.0
Toth *et al*., 2014	58 (34 CRC, 24 NED)	88.2% (30/34)	91.7% (22/24)	10.59	0.13	82.50	2/3	Epi proColon 2.0
Kang *et al*.^21^	132 (80 CRC, 52 NED)	75.0% (60/80)	98.1% (51/52)	39.00	0.25	153.00	2/3	Epi proColon 2.0
He *et al*., 2014	281 (76 CRC, 69 Polyps, 136 NED)	71.1% (54/76)	95.6% (196/205)	16.18	0.30	53.45	2/3	Epi proColon 2.0
Jin *et al*.^23^	226 (135 CRC, 91 control)	74.8% (101/135)	96.7% (88/91)	22.69	0.26	87.14	2/3	Epi proColon 2.0
Yu *et al*., 2015	123 (70 CRC, 53 NED)	81.4% (57/70)	86.8% (46/53)	6.17	0.21	28.81	2/3	Epi proColon 2.0
Ørntoft *et al*., 2015	278 (128 CRC, 150 NED)	72.7% (93/128)	82.0% (123/150)	4.04	0.33	12.10	1/3	Epi proColon 2.0
		58.6% (75/128)	95.3% (143/150)	12.47	0.43	28.91	2/3	
Li *et al*., 2015	138 (91 CRC, 47 NED)	72.5% (66/91)	91.5% (43/47)	8.53	0.30	28.38	1/1	SensiColon
He *et al*., 2015	100 (50 CRC, 50 NED)	76.0% (38/50)	96.0% (48/50)	19.00	0.25	76.00	1/1	Research kit
Ding *et al*.^25^	182 (82 CRC, 100 NED)	73.2% (60/82)	96.0% (96/100)	18.30	0.28	65.45	2/3	Epi proColon 2.0
Wu *et al*.^10^	586 (291 CRC, 295 NED)	76.6% (223/291)	95.9% (283/295)	18.84	0.24	77.34	1/1	SensiColon
Song *et al*.^11^	859 (369 CRC, 490 NED)	82.4% (303/369)	82.0% (402/490)	4.58	0.21	20.97	1/3	Epi proColon 2.0
		75.1% (277/369)	97.1% (476/490)	25.90	0.26	102.37	2/3	

PLR = positive likelihood ratio, NLR = negative likelihood ratio, OR = odds ratio, CRC = colorectal cancer, NED = no evidence of diseases, AA = advanced adenoma, NAA = non-advanced adenoma, SP = small polyps, LDT = laboratory developed test.

### The blood SEPT9 gene methylation exhibits adequate sensitivity and specificity in CRC detection and screening

Table 1 summarized the sensitivity, specificity, PLR, NLR, OR, algorithm and kits used in each study. The sensitivity and specificity of the cohort or case-control study were affected by study design, population, selection of cases, choice of kits and algorithm, etc. Generally speaking, the sensitivity of these studies ranged from 48.2% to 95.6%, with the specificity ranged from 79.1% to 99.1% (Table 1). The latest commercialized SEPT9 assay, the Epi proColon 2.0, exhibited a higher sensitivity at 71.1–95.6%, and maintained high specificity at 81.5 to 99% (Table 1). The effect of different algorithm can be observed in studies with multiple algorithms applied, and it will be discussed further in this article.

The PLR of the 25 studies ranged from 2.87 to 73.00, exhibiting a high ratio between true positive and false positive rate and suggesting a high probability of true positive when a test result is positive. The NLR of the 25 studies ranged from 0.05 to 0.57, exhibiting a high ratio between false negative and true negative rate and suggesting a high probability true negative when a test result is negative. The OR of the 25 studies ranged from 5.67 to 349.63, indicating that the SEPT9 methylation is a high-risk factor and has diagnostic significance for CRC. PLR, NLR and OR exhibited a big variation among all studies. This may be due to different design of studies, since various inclusion of CRC cases, non-CRC colonic diseases, and normal controls in case-control, cohort or screening studies can greatly affect the positive and negative rate. This may also be due to the difference in kit performance, as those early stage research kits and commercialized kits exhibited lower detection capability than later improved kits.

Currently, the PRESEPT study in the only screening study performed in average-risk population from 50 to 75 years old. The sensitivity reported (48.2%) using 1/2 algorithm was apparently lower than those reported in previous cohort or case-control studies. In a later report by Potter and colleagues^9^, triplicate PCRs were performed using samples from the same study. The sensitivity increased to 68.2% and the specificity decreased to 80.0%. The US FDA approved the Epi proColon, the commercialized SEPT9 assay, based on the data from the PRESEPT study with 1/3 algorithm^7, 9^. Apart from the screening in average-risk population, the SEPT9 assay was also used in the opportunistic screening for high-risk population. In one study performed in four northern Chinese hospitals (RESEPT study) using the SensiColon assay, the SEPT9 assay exhibited a sensitivity of 76.6% with a specificity of 95.9% at a total positive rate of 25.8%^10^. In another recent opportunistic screening study using the Epi proColon 2.0 CE kit, the SEPT9 assay exhibited a sensitivity of 75.1% with a specificity of 95.1%^11^.

### The choice of algorithm affects the performance of the SEPT9 assay

Table 1 lists the four algorithms currently used in studies of SEPT9 assay. The positive test results were determined by one positive count out of three PCRs (1/3 algorithm), one positive count out of two PCRs (1/2 algorithm), two positive counts out of three PCRs (2/3 algorithm), or one positive count out of one PCR (1/1 algorithm). It can be clearly seen that a majority of studies performed 1/3 or 2/3 algorithm, while 1/2 and 1/1 algorithm were also used for some studies.

**Figure 2:**
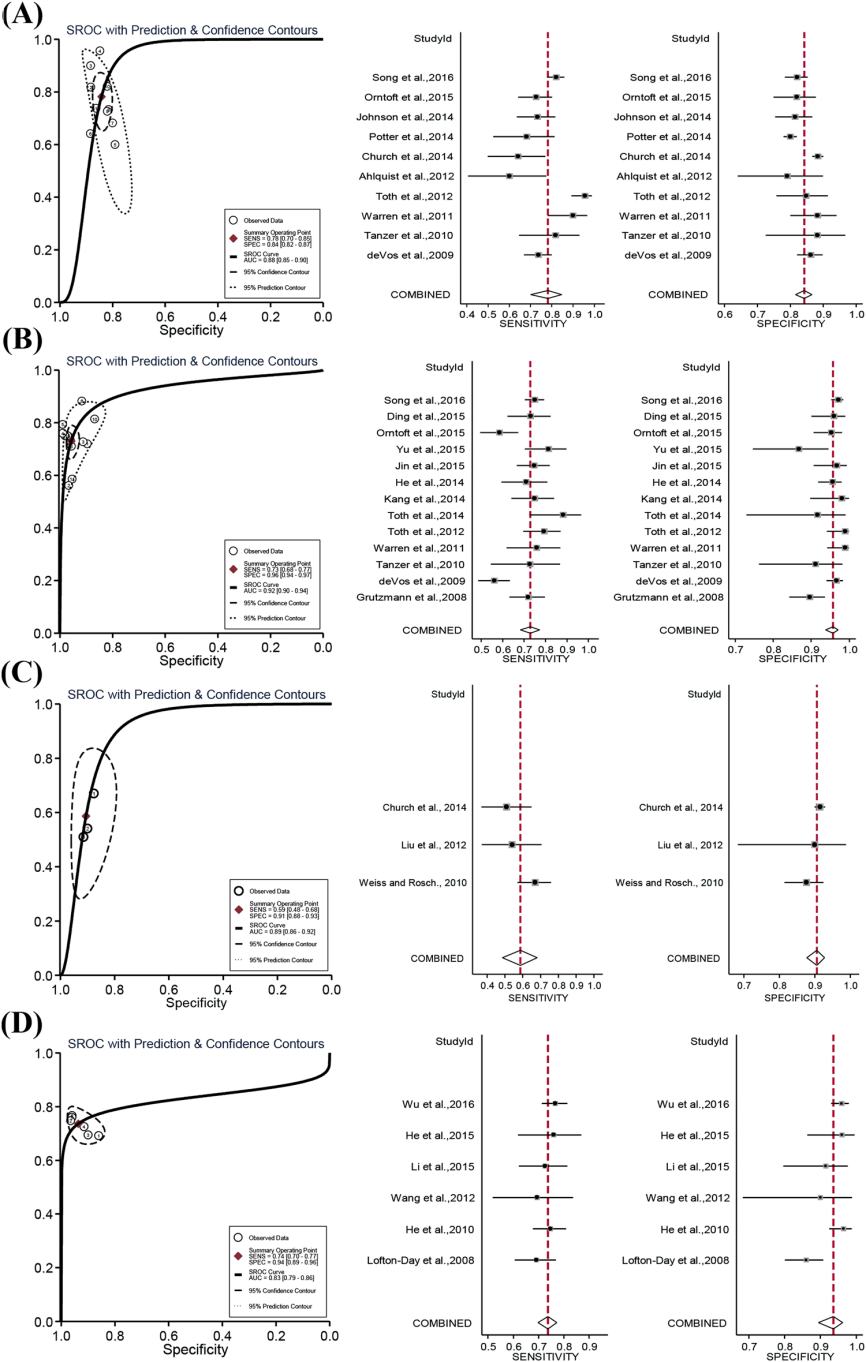
The sROC and forest plots of summary estimates of sensitivity and specificity of SEPT9 assay with various algorithms. The sROC curves and the Forest plots are shown for 1/3 (**A**), 2/3 (**B**), 1/2 (**C**) and 1/1 (**D**) algorithms. The sROC curves represent the ROC plot of the hierarchical summary estimates of sensitivity and specificity for SEPT9 assay with 95% confidence and prediction ellipses for various algorithms. In the Forest plot, the sensitivity or specificity for each study was plotted as solid squares with bars indicating 95% confidence interval. The red lines indicate the pooled estimates of sensitivity or specificity. The rhombus indicates the 95% confidence interval of the pooled estimates.

In order to compare the performance of SEPT9 assay at various algorithms, study data from each algorithm were pooled and meta-analyzed. As shown in Fig. 2, 1/3 algorithm exhibited the best sensitivity (0.78) with lowest specificity (0.84) among all algorithms (Fig. 2A), while 2/3 algorithm exhibited the highest specificity (0.96) (Fig. 2B). The sensitivity and specificity of 2/3 and 1/1 algorithm (Fig. 2B and D) were very similar (sensitivity: 0.73 vs 0.74, specificity: 0.96 vs 0.94). 1/2 algorithm (Fig. 2C) exhibited the lowest sensitivity (0.59) with satisfactory specificity (0.91). The area under the curve (AUC) showed very similar values for 1/3, 2/3 and 1/2 algorithm, while the AUC for 1/1 algorithm appeared to be slightly lower than others. It can be observed that the 1/3 algorithm provides the best sensitivity at the price of lower specificity, while the 2/3 algorithm provides the best balance between sensitivity and specificity.

### SEPT9 assay is effective for the detection of early-stage CRC

**Figure 3:**
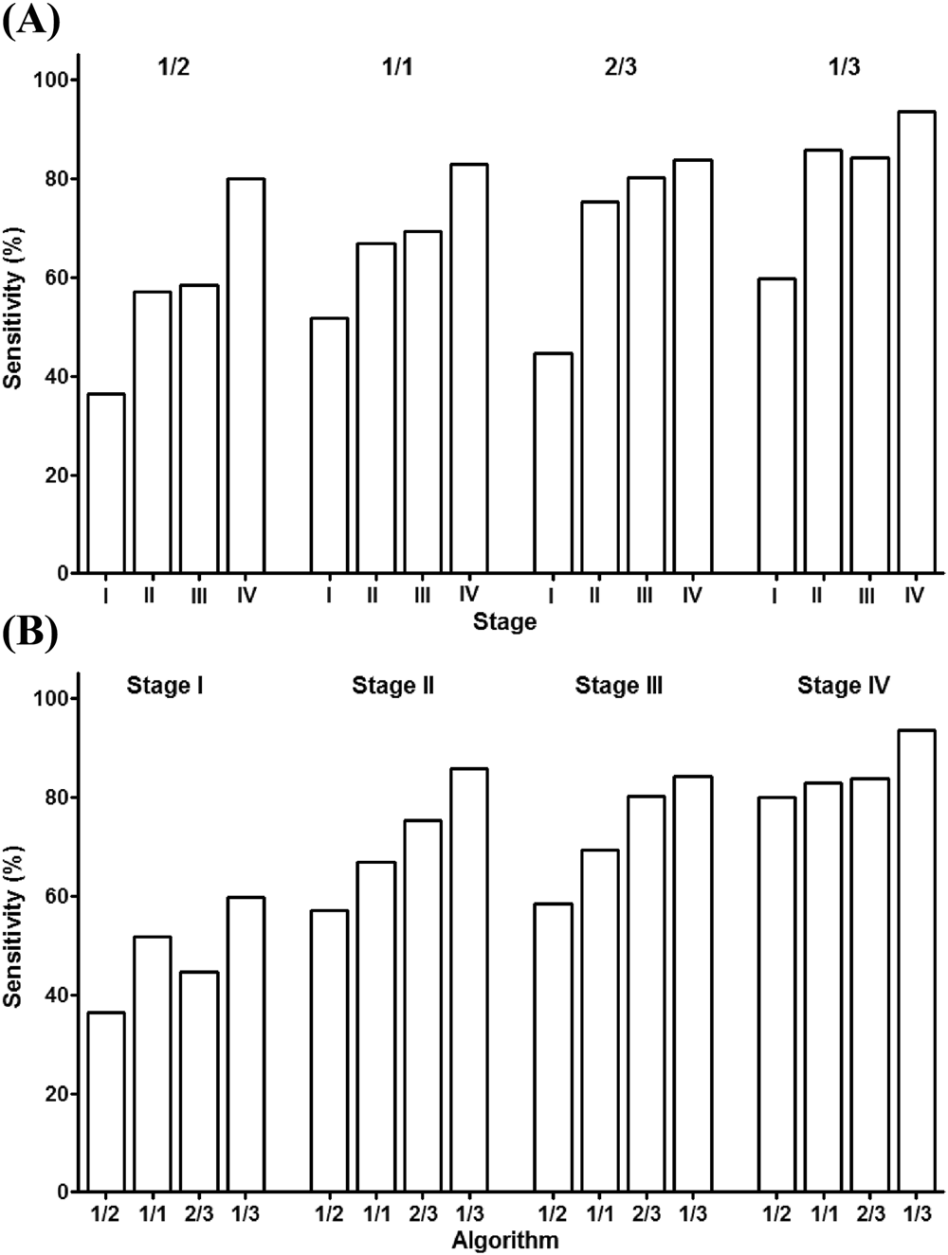
The stage-dependent sensitivity for each CRC stage with 1/3, 2/3, 1/2 or 1/1 algorithm. Panel A compares the sensitivity for each CRC stage at each algorithm. It is clear that the sensitivity increased with the elevation of stages, no matter what algorithm was applied. Panel B compares the sensitivity calculated from each algorithm at each CRC stage. Generally, 1/3 algorithm exhibited the highest sensitivity, followed by 2/3, 1/1 and 1/2 algorithms, no matter what stage was compared.

Although SEPT9 assay exhibited overall high sensitivity and specificity in detecting CRC, the ability of detecting early-stage CRC is more important than later stages of CRC, since early detection can ensure early intervention to enhance the cure rate and reduce mortality. Stage I and II are regarded as early stages in this article. Table 2 summarizes the stage-related detection rate of all available studies and the pooled data categorized by the algorithm used in these studies. The data in Table 2 is plotted in Fig. 3 to compare the detection rate at each stage (Fig. 3A) and the effect of algorithm on stage-dependent detection rate (Fig. 3B). It can be seen from Table 2 and Fig. 3 that there is a clear trend in which the detection rate increases with the escalation of clinical stage, indicating that the level of SEPT9 methylation had a correlation with the degree of malignancy, regardless of the algorithm. Furthermore, the algorithm exhibited a clear effect on the sensitivity for every stage (Fig. 3B). The sensitivity with various algorithm can be ranked as 1/3 > 2/3 > 1/1 > 1/2 from highest to lowest.

**Table 2:** Sensitivity for each CRC stage with 1/3, 2/3, 1/2 or 1/1 algorithm.

Algorithm	Study	Stage			
		I	II	III	IV
1/3	deVos *et al*.^26^	52.6% (10/19)	75.0% (30/40)	77.8% (21/27)	100.0% (4/4)
	Warren *et al*., 2011	71.4% (5/7)	90.3% (28/31)	100.0% (7/7)	100% (5/5)
	Ahlquist *et al*., 2012	57.1% (4/7)	57.1% (4/7)	37.5% (3/8)	87.5% (7/8)
	Toth *et al*., 2012	84.0% (21/25)	100.0% (14/14)	100.0% (35/35)	100.0% (18/18)
	Johnson *et al*.^13^	61.5% (16/26)	80.0% (16/20)	65.2% (15/23)	92.3% (12/13)
	Ørntoft *et al*., 2015	37.1% (13/35)	91.4% (32/35)	76.7% (23/30)	89.3% (25/28)
	Song *et al*.^11^	64.3% (27/42)	87.6% (92/105)	87.8% (115/131)	93.3% (14/15)
	**Overall**	**59.6% (96/161)**	**85.7% (216/252)**	**84.2% (219/261)**	**93.4% (85/91)**
2/3	Grutzmann *et al*., 2008	50.0% (11/22)	69.4% (25/36)	79% (42/53)	91% (10/11)
	deVos *et al*.^26^	26.3% (5/19)	60.0% (24/40)	66.7% (18/27)	75.0% (3/4)
	Toth *et al*., 2012	60.0% (15/25)	92.8% (13/14)	81.6% (31/35)	77.8% (14/18)
	Kang *et al*.^21^	48.0% (12/25)	82.6% (19/23)	93.1% (27/29)	66.7% (2/3)
	He *et al*., 2014	35.7% (5/14)	81.0% (17/21)	79.3% (23/29)	80.0% (8/10)
	Jin *et al*.^23^	66.7% (12/18)	82.6% (19/23)	84.1% (37/44)	100.0% (5/5)
	Ørntoft *et al*., 2015	17.1% (6/35)	74.3% (26/35)	63.3% (19/30)	85.7% (24/28)
	Ding *et al*.^25^	50.0% (1/2)	53.3% (16/30)	87.5% (35/40)	80.0% (8/10)
	Song *et al*.^11^	54.8% (23/42)	82.9% (87/105)	78.6% (103/131)	86.7% (13/15)
	**Overall**	**44.6% (90/202)**	**75.2% (246/327)**	**80.1% (335/418)**	**83.7% (87/104)**
1/2	Church *et al*.^7^	36.4% (8/22)	57.1% (8/14)	58.3% (7/12)	80.0% (4/5)
	**Overall**	**36.4% (8/22)**	**57.1% (8/14)**	**58.3% (7/12)**	**80.0% (4/5)**
1/1	Lofton-Day *et al*.^3^	30.0% (6/20)	56.3% (18/32)	44.7% (21/47)	67.7% (21/31)
	Li *et al*., 2015	40.0% (2/5)	65.5% (19/29)	88.2% (15/17)	88.9% (16/18)
	Wu *et al*.^10^	64.9% (24/37)	72.7% (48/66)	79.3% (65/82)	93.9% (31/33)
	**Overall**	**51.6% (32/62)**	**66.9% (85/127)**	**69.2% (101/146)**	**82.9% (68/82)**

SEPT9 assay generally exhibited the highest detection rate for all stages of CRC with 1/3 algorithm (Fig. 3B). The detection rate for stage I and II reached 59.6% and 85.7%, respectively (Table 2), representing a very high detection rate for early-stage CRC among current *in-vitro* diagnostic methods. The SEPT9 assay with 2/3 or 1/1 algorithm detected approximately half of stage I and 70% of stage II CRC, respectively, which was also satisfactory for early CRC detection (Table 2, Fig. 3B). Apparently, for CRC screening aiming at early-stage cancer detection, algorithms with high sensitivity should be adopted. However, false positive detection will also increase with higher sensitivity algorithms. These data clearly show that the SEPT9 assay is effective for early stage CRC detection.

### The Performance of the SEPT9 assay is superior to CEA and the FIT tests in the screening of symptomatic population

**Figure 4:**
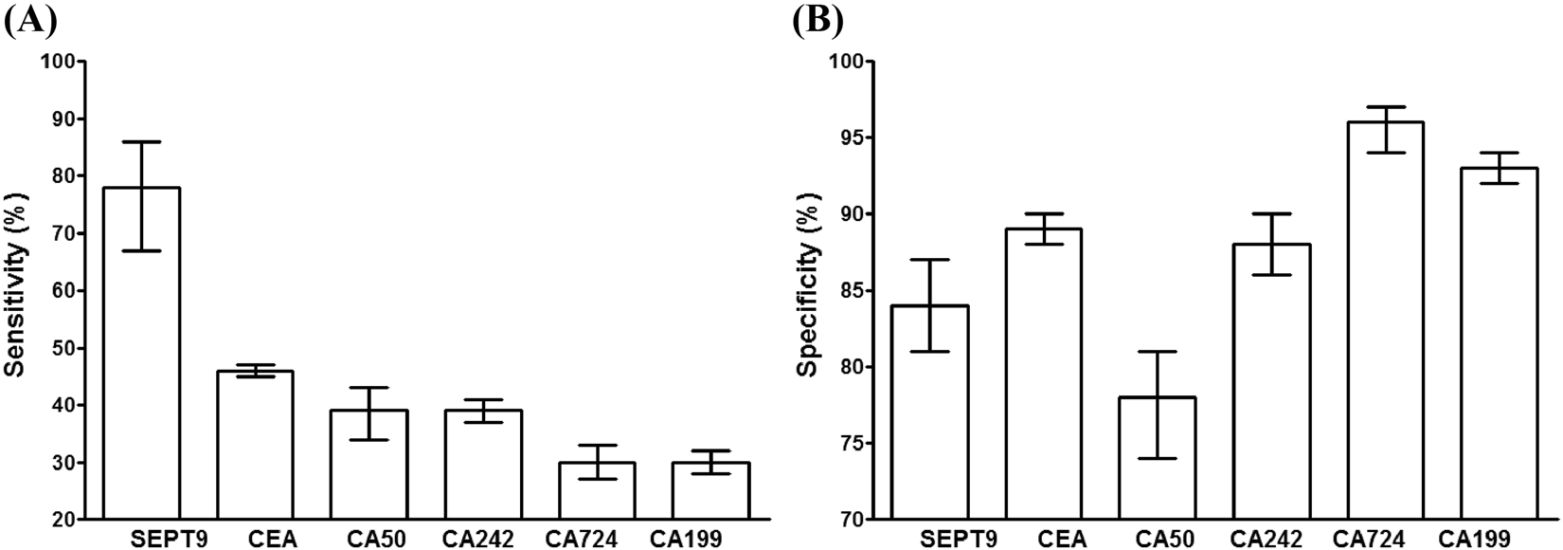
The sensitivity and specificity of the SEPT9 assay were superior to serum protein markers in symptomatic population. The sensitivity of the SEPT9 assay in CRC screening or detection appeared to be much higher than that of the CEA, CA199, CA242, CA50 and CA724, as shown in panel A. In contrast, its specificity was at the same range as these serum protein markers (panel B). Bars indicate 95% confidence interval.

In order to further evaluate the performance of the SEPT9 assay in CRC screening and diagnosis, the sensitivity and specificity of the assay was compared with the current clinically used serum markers (CEA, CA50, CA242, CA724 and CA199)^32^ in the screening of symptomatic population. As 1/3 algorithm was recommended by the US FDA as the interpretation method, the pooled data from 1/3 algorithm of the assay was used for comparison. The sensitivity and specificity of these tests were meta-analyzed by reviewing the study on the performance of each individual test on CRC detection. The Forest plot was made for each serum marker and the estimated sensitivity and specificity were analyzed for comparison with the SEPT9 assay (Fig. 4A and B). It can be clearly seen from Fig. 4A and B that the sensitivity of the SEPT9 assay (78%) in CRC detection was much higher than any of the five serum markers, and its specificity (84%) was at the same range as these serum markers, including the most commonly used CRC serum marker, CEA. The detailed data for the serum markers was summarized and analyzed in Supplementary Figure 2 (CEA), 3 (CA50), 4 (242), 5 (CA724) and 6 (CA199), respectively.

As FIT is a test widely used for CRC screening in symptomatic population, three studies compared the performance of FIT with that of the SEPT9 assay side by side. Table 3 summarized the sensitivity and specificity of FIT and the SEPT9 assay in the three studies. It can be seen from the pooled data that the SEPT9 assay exhibited significantly higher sensitivity than the FIT test (75.6% *vs* 67.1%, p < 0.05), while they showed essentially identical specificity. It appeared that the performance of the SEPT9 assay in screening of symptomatic population is better than that of the FIT test.

**Table 3:** Performance comparison between the SEPT9 and FIT assays in symptomatic population.

Publication	Sensitivity		Specificity	
	SEPT9	FIT	SEPT9	FIT
Johnson *et al*.^13^	73.3%	68.0%	81.5%	97.4%
	(74/101)	(66/97)	(163/200)	(188/193)
Jin *et al*.^23^	74.8%	58.0%	87.4%	82.4%
	(101/135)	(40/69)	(298/341)	(89/108)
Wu *et al*.^10^	77.0%	74.6%	94.2%	N/A
	(181/235)	(53/71)	(697/740)	N/A
**Overall**	**75.6%**	**67.1%**	**90.4%**	**92.0%**
	**(356/471)**	**(159/237)**	**(1158/1281)**	**(277/301)**

### The SEPT9 assay is less potent than the FIT and the FIT-DNA tests in the screening of asymptomatic population

**Figure 5:**
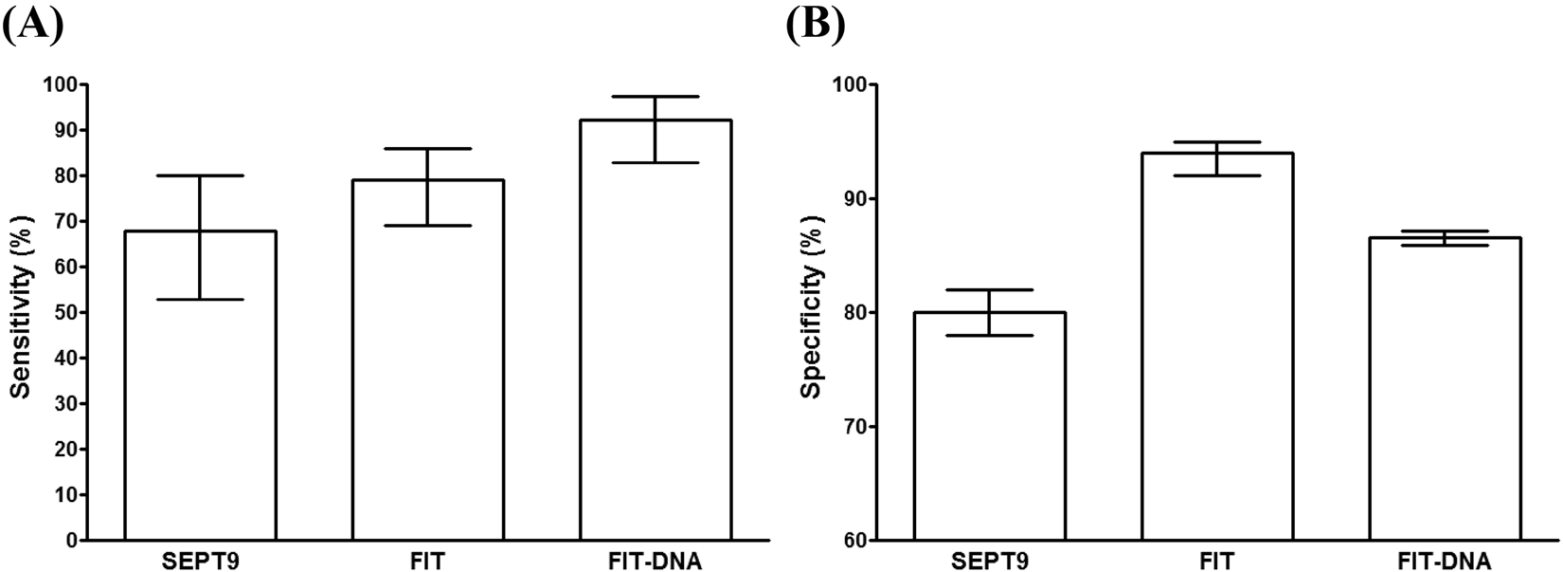
The sensitivity and specificity of the SEPT9 assay were lower than FIT and FIT-DNA tests in asymptomatic population. The sensitivity of the SEPT9 assay in CRC screening in asymptomatic average-risk population appeared to be lower than that of the FIT and FIT-DNA tests, as shown in panel A. Similarly, its specificity was also lower than the other two tests (panel B). Bars indicate 95% confidence interval.

Currently, the PRESEPT study^7, 9^ is the only study investigated the performance of the blood SEPT9 assay in average-risk asymptomatic population, we therefore compared the data from this report with data from FIT^33^ and FIT-DNA test^34^ in the same type of population. It can be clearly seen from Fig. 5A and B that SEPT9 exhibited lower sensitivity (68.0% for the SEPT9 assay, compared with 79.0% for FIT and 92.3% for FIT-DNA test) and lower specificity (80.0% for the SEPT9 assay, compared with 94.0% for FIT and 86.6% for FIT-DNA test) than FIT or FIT-DNA test. The performance of the blood SEPT9 assay in asymptomatic population screening appeared to be lower than that of the FIT and FIT-DNA tests. However, the SEPT9 assay exhibited better compliance than FIT test. One recent study^35^ showed that 63% of subjects recommended for CRC screening refused colonoscopy screening. 97% of subjects refusing colonoscopy accepted a noninvasive screening test, in which 83% chose the Septin9 blood test and 15% chose FIT test. It is clear from the study that patients prefer SEPT9 test than colonoscopy and FIT^35^.

## Discussion

It is notable that the screening sensitivity of 48.2% reported in the PRESEPT study was much lower than those reported in previous cohort or case-control studies. This can be explained from three aspects. Firstly, duplicate PCR reactions, instead of triplicate PCRs, were used in this study. The sensitivity in this study was obtained from at least one positive reaction out of two PCRs, instead of three PCRs, therefore, the chance to detect abnormally methylated SEPT9 DNA was lower than those previous studies performing three PCRs. Secondly, the study setting was different from those previous studies. The PRESEPT study aimed at screening of asymptomatic average-risk population between 50 and 75 years old. CRC patients found in the asymptomatic population are more likely to be those with early-stage CRC. As the sensitivity in early-stage CRC appeared to be lower than the overall sensitivity from all stages, the sensitivity from the asymptomatic population tends to be lower than those with symptomatic population, i.e. CRC subjects recruited from hospitals in those case-control or cohort studies. Thirdly, the reaction system in different studies varied, and this may explain why 1/1 algorithm exhibited higher performance than 1/2 algorithm. The PCR reaction from Epi proColon series product used 30 µl reaction system, however, is was reported that the latest SensiColon product used 60 µl reaction system with doubled amount of DNA template^10^. This allowed higher chance of methylated DNA detection, although only one PCR reaction was performed.

The PRESEPT study represents the scenario in the real life CRC screening setting and therefore has better guiding significance than case control studies. The application of three PCRs was based on the consideration of identifying as many cancer patients as possible in the screening background. It appears that the sensitivity reported in the study using 1/3 algorithm (68.2%) was much better than that obtained from 1/2 algorithm^9^. Therefore, 1/3 algorithm was accepted by the US FDA for the approved product.

Apart from the screening of average-risk population, the SEPT9 assay was also used in the opportunistic screening of high-risk population, in which the chance of identifying positive subjects is much higher than that in average-risk population, as this screening is commonly performed in hospital environment. Therefore, the positive rate, sensitivity and specificity are related to specific population in a screening. The SEPT9 assay exhibited high sensitivity and specificity in opportunistic screening^10^. These parameters appeared to be higher than those in PRESEPT study, as the case composition in this opportunistic screening was quite different from that in REPSEPT study.

A distinct feature of the SEPT9 assay is that most studies performed multiple PCRs to enhance test sensitivity. This is because SEPT9 assay is designed for detecting trace amount of methylated SEPT9 gene copies in strong background of unmethylated SEPT9 DNA, and the amount of detectable methylated SEPT9 DNA can be as low as 7.8 pg/ml, equivalent to 1–2 copies of genome DNA^9^. This leads to the question of interpreting the PCR data when some of the PCR reactions show positive while others show negative results. Due to the application of different algorithms in data analysis, sensitivity and specificity vary with different methods of interpretation.

The choice of algorithms is based on the specific needs in a test. Two aspects need to be considered before a method can be properly chosen. One is to exclude healthy negative population, which requires high specificity, and the other is to identify as many real patients as possible to enhance the disease detection rate, which requires high sensitivity. These two aspects are normally against each other, as they are two factors at the ends of a teeterboard. As the 1/3 algorithm showed the best sensitivity while the 2/3 algorithm showed the best specificity, the choice of 1/3 algorithm or 2/3 algorithm depends on the purpose of a study. If detecting cancer is the main purpose, such as that in a screening test, 1/3 algorithm can be used to maximize the number of patients detected. The situation of false positive subjects can be confirmed by further diagnostic method, such as colonoscopy. In contrast, if the main purpose is to exclude healthy subjects to ensure low misdiagnosis rate, 2/3 algorithm can be used, and routine screening program should be implemented to minimized the ratio of missed cancer patients.

As the SEPT9 assay was shown to detect early-stage CRC with high sensitivity, it has great advantages over serum protein markers in CRC early detection. CEA is the most commonly used markers for CRC detection, however, due to its low sensitivity for early-stage CRC, it is more widely used in post-surgery monitoring of CRC recurrence and therapeutic effects, rather than a screening marker. SEPT9 gene methylation appeared to be the best blood-based single marker for CRC screening and early detection so far.

Although the SEPT9 assay did not exhibit similar sensitivity and specificity compared with FIT and FIT-DNA in asymptomatic population screening, it is a competitive option for CRC screening and early detection, as it has been shown to have higher compliance in CRC screening than FIT and colonoscopy^35^. A good screening test should be one that not only has high sensitivity and specificity, but also has high uptake rate by population.

## Conclusions

The SEPT9 assay is the first blood-based test aiming at ctDNA detection for CRC screening and early detection. It shows a high sensitivity and specificity in CRC screening and early detection. The choice of algorithm is based on the needs for a test. Algorithm with high sensitivity (1/3) can be used in screening, while algorithm with high specificity (2/3 or 1/1) can be used in CRC early detection for diagnostic purpose to exclude normal subjects. The SEPT9 assay exhibited better performance than protein markers and FIT test in symptomatic population, while showed lower sensitivity and specificity than the FIT and the FIT-DNA tests in asymptomatic population. However, it provides an effective option for patients who have low compliance with FIT or colonoscopy.

## Methods

All literature search, selection, data extraction, study quality assessment, and data analysis were performed based on the rules, guidelines or recommendations from the preferred reporting items for systematic reviews and meta-analyses (PRISMA) and the quality assessment for diagnostic accuracy studies (QUADAS)^8, 30, 31, 36, 37^. Relevant softwares were used for the above process and data analysis, including Review Manager 5.2 (The Cochrane Collaboration, London, UK), Stata 14.0 (StataCorp LP, TX, USA), MetaDisc 1.4 (Unit of Clinical Biostatistics team of the Ramóny Cajal Hospital, Madrid, Spain) and PRISM 5.0 (GraphPad Software, Inc., La Jolla, CA, USA).

### Search strategy

The Ovid MEDLINE,EMBASE, CBMdisc (China Biology Medicine disc) and CJFD (Chinese Journal Full—text Database) database were searched using the key words ‘SEPT9′, or ‘septin 9′, and ‘colorectal cancer’ or ‘colorectal carcinoma’ to identify all relevant studies. 215 studies were identified from MEDLINE and EMBASE, and 151 relevant articles were identified from CMBdisc and CJFD (Fig. 1).

### Study inclusion and exclusion criteria

The aim of the study selection is to identify the studies that are clinical studies evaluating the performance of the SEPT9 assay using blood samples from human subjects. (Fig. 1). Duplicates from all databases were excluded and 180 studies remained. In the next screening, a total of 104 articles, including letters, reviews, meta-analysis and guidelines (18 in MEDLINE and EMBASE, 61 in CBMdisc and CJFD), basic research studies (3 in MEDLINE and EMBASE, 7 in CBMdisc and CJFD) and articles irrelevant to mSEPT9 detection assays (3 in MEDLINE and EMBASE, 12 in CBMdisc and CJFD) were excluded. In the following eligibility screening, a further 48 articles were excluded, including studies that were not using plasma or serum samples (16 in MEDLINE and EMBASE, 5 in CBMdisc and CJFD), studies that were not detecting gene methylation (9 in MEDLINE and EMBASE, 18 in CBMdisc and CJFD). Finally, 28 studies were included in the qualitative synthesis, and studies that did not have statistically significant number of CRC or non-CRC cases included (3 in CBMdisc and CJFD), and 25 studies were included in the quantitative synthesis for this meta-analysis.

### Data extraction

A standardized data abstraction form was developed, and key elements related to test parameters were collected by two independent reviewers. The study design, type of study (case-control, cohort or screening), sample types, sample size (number of cases, controls, males, females), subject age distribution, algorithm and gold standard for diagnosis were first examined and recorded to ensure the validity of a study. The test sensitivity, specificity and positivity rate with detailed positive, negative and total numbers of cases were then collected. The PLR, NLR and OR were calculated based on these numbers.

### Study quality and risk of bias assessment

**Figure 6:**
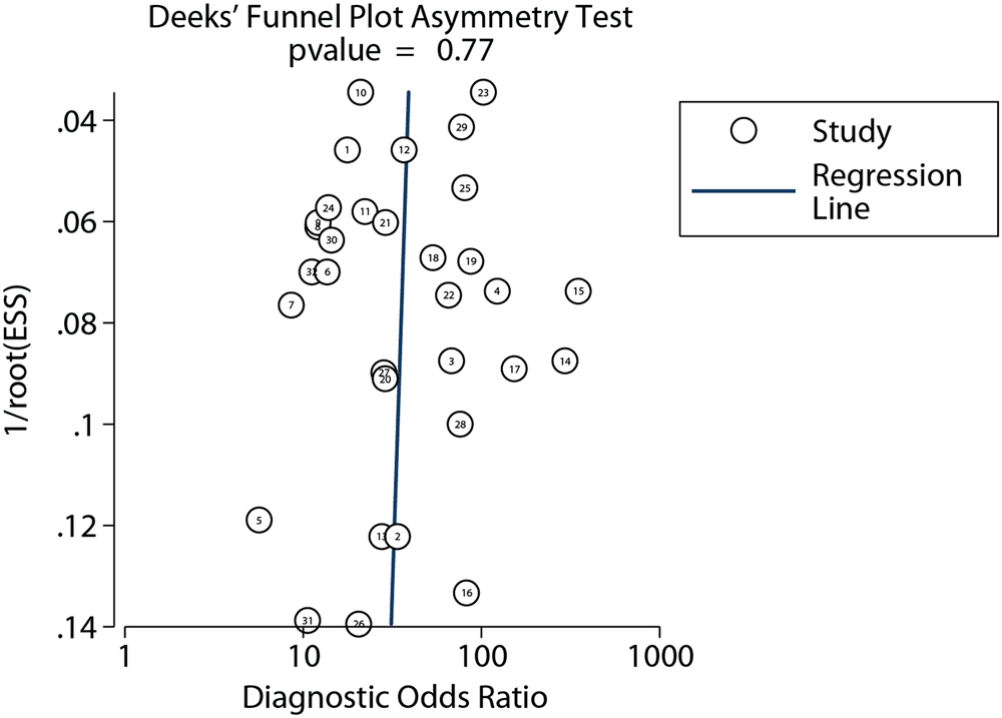
Deeks’ funnel plot asymmetry test for all studies included in this meta-analysis.

The quality of the included reports was assessed using the QUADAS system^8^ by Review Manager 5.2 software. The methodological quality of the studies with focuses on the risk of bias and applicability was systematically assessed, as shown in Supplementary Figure 1. All items in the PRISMA checklist was assessed and finished, as shown in Supplementary Table 1. Most studies were cohort or case-control studies, and only three of them were prospective screening studies. Therefore, some bias inevitably appeared in cohort or case-control studies. Although most of them clearly describe the patient selection criteria and procedure, and the number of CRC cases was comparable with the number of controls, a few of them were lack of description of selection criteria, or had unbalanced number of cases against controls, and these may lead to the risk of bias. As the kits from Epigenomics, Inc. were used for a majority of studies, the cutoff values were identical for most studies, and the index test (marker test) and the reference test were performed parallel. Finally, colonoscopy and subsequent pathological examination were used as the gold standard for cancer or normal subject determination for all studies, and this ensured the quality of data. The overall bias of the included studies was tested using the Deeks’ funnel plot (Fig. 6), and the P value of 0.77 indicates that the distribution of studies is symmetric and there is no systematic bias across all studies analyzed in this study.

### Subgroup analyses and definition of diagnostic outcomes

All studies were divided into four subgroups based on the algorithm used in the interpretation of multiple qRT-PCR or high-resolution melting (HRM) data. If the final positive test result was determined from at least one positive count out of three repeats, the study was categorized into the 1/3 algorithm group, and if the final positive test result was determined from at least two positive counts out of three repeats, the study was categorized into the 2/3 algorithm group. Similarly, if the final positive test result was determined from at least one positive count out of two repeats, the study was categorized into the 1/2 algorithm group, while if the final positive test result was determined from only one reaction, the study was categorized into the 1/1 algorithm group. The sensitivity, specificity, PLR, NLR and OR were calculated based on the algorithm, and parameters for each algorithm were calculated by pooling the data from studies in the same algorithm group. The stage-dependent sensitivity was calculated based on the number of positive cases and the total number of cases for a certain stage and the algorithm used in the study.

**Figure 7:**
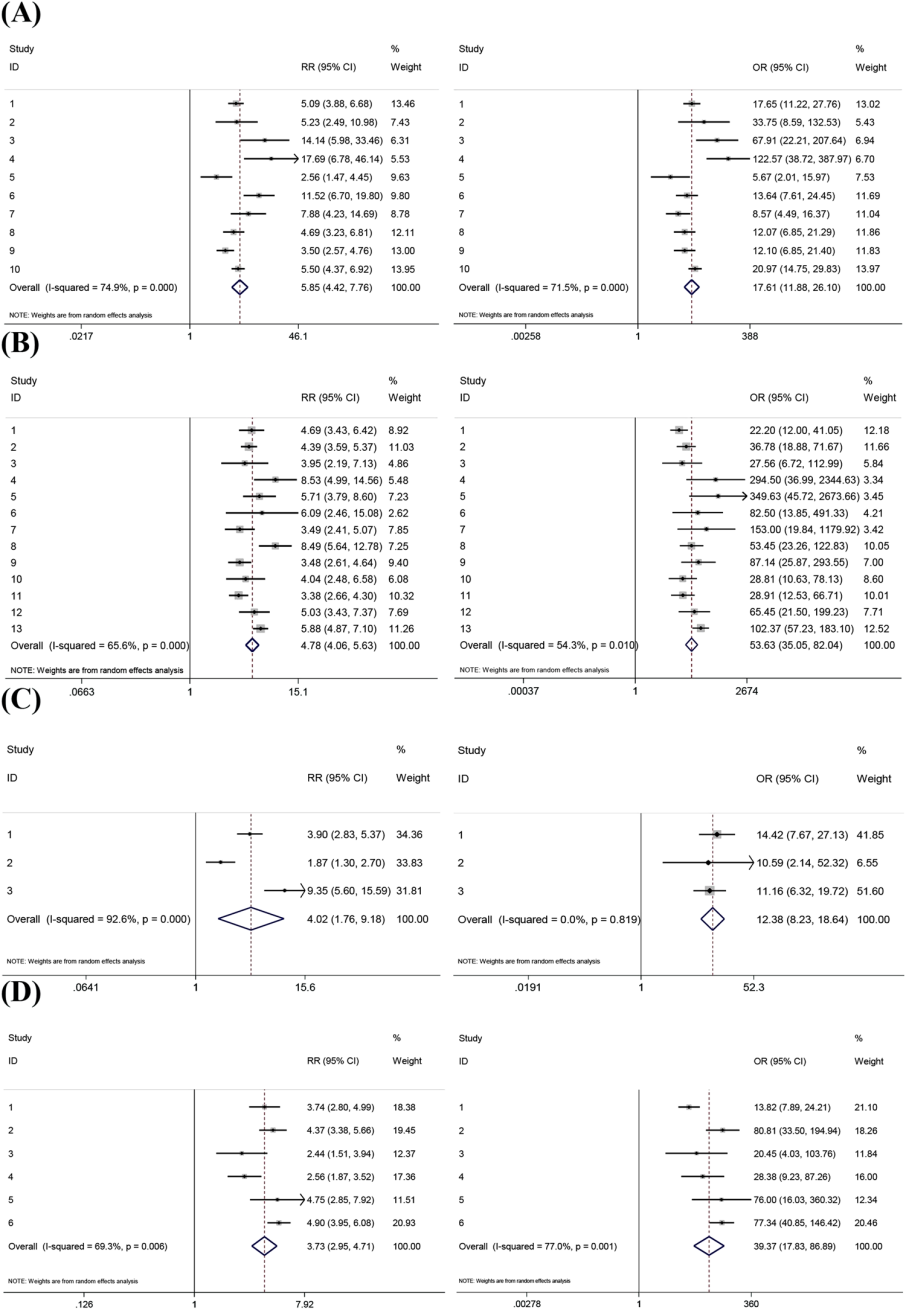
Heterogeneity analysis is performed for studies in all four algorithms in SEPT9 assay data interpretation. RR and OR are used as indicators for heterogeneity in 1/3 algorithm (**A**), 2/3 algorithm (**B**), 1/2 algorithm (**C**) and 1/1 algorithm (**D**). Left figures in each panel represent RR analysis and right figures in each panel represent OR analysis.

The heterogeneity of the studies was analyzed based on four algorithms used for SEPT9 assay interpretation, and RR (relative risk) and OR (odds ratio) values were calculated to show the heterogeneity in all four algorithms. It can be seen from Fig. 7 that all RR analysis (the left figure in each panel) showed high I^2^ values (69.3% to 92.6%) with very small P values, indicating the existence of heterogeneity in studies for all four algorithms. The OR analysis (the right figure in each panel) showed high I^2^ values (54.3–77.0%) with very small P values in 1/3. 2/3 and 1/1 algorithm, suggesting the presence of heterogeneity, while did not show heterogeneity for 1/2 algorithm. Taken together, it can be suggested that heterogeneity was present among studies in all algorithms, and the random effect model should be used for analysis.

### Statistical analysis

The sROC curves for each algorithm were simulated, and the Forest plot for each algorithm and the relevant statistics (including estimated sensitivity, specificity, area under curve (AUC), 95% confidence interval, 95% confidence contour, and 95% prediction contour) were performed using the Stata 14.0 software. The Forest plot for the five serum protein markers and relevant statistics were performed using the Meta-disc 1.4 software (Supplementary Figures 2– 6). The estimates and 95% confidence interval for all data in histograms were calculated and plotted using the PRISM 5.0 software. The comparison of ratio parameters was performed using the χ^2^ test, and p < 0.05 was regarded as a significant difference.

## Electronic supplementary material

Supplementary information
