# Supplementary material for: The performance of the SEPT9 gene methylation assay and a comparison with other CRC screening tests: A meta-analysis
Source: Sci Rep. 2017 Jun 8;7:3032. doi: 10.1038/s41598-017-03321-8 (PMC5465203; doi:10.1038/s41598-017-03321-8)
Supplement: Supplementary file 1 — Supplementary information [file 41598_2017_3321_MOESM1_ESM.pdf]

The performance of the SEPT9 gene methylation assay and a comparison with other CRC screening tests: A meta-analysis

Lele Song[1,2,*], Jia Jia[1,3], Xiumei Peng[4,5], Wenhua Xiao[5], Yuemin Li[1,*]

1.Department of Radiotherapy, The Chinese PLA 309th Hospital, Beijing 100091, P.R. China

2.BioChain (Beijing) Science and Technology, Inc., Beijing 100176, P.R. China.

3, Department of Graduate, Hebei North University, Zhangjiakou, Hebei, P.R. China

4, Medical School of Chinese PLA and Chinese PLA General Hospital, Beijing, P.R. China

5, Department of Oncology, First Affiliated Hospital of Chinese PLA General Hospital, Beijing, P.R. China

*Corresponding Author:

Yuemin Li, M.D., Department of Radiotherapy, The Chinese PLA 309th Hospital, No. 17, Heishanhu Road, Haidian District, Beijing 100091, P.R. China, email: liyuemin224@sina.com, Tel: 86-10-66775222

Lele Song, M.D., Ph.D., Department of Radiotherapy, The Chinese PLA 309th Hospital, No. 17, Heishanhu Road, Haidian District, Beijing 100091, P.R. China, email: songlele@sina.com, Tel: 86-13240149188

Supplementary information

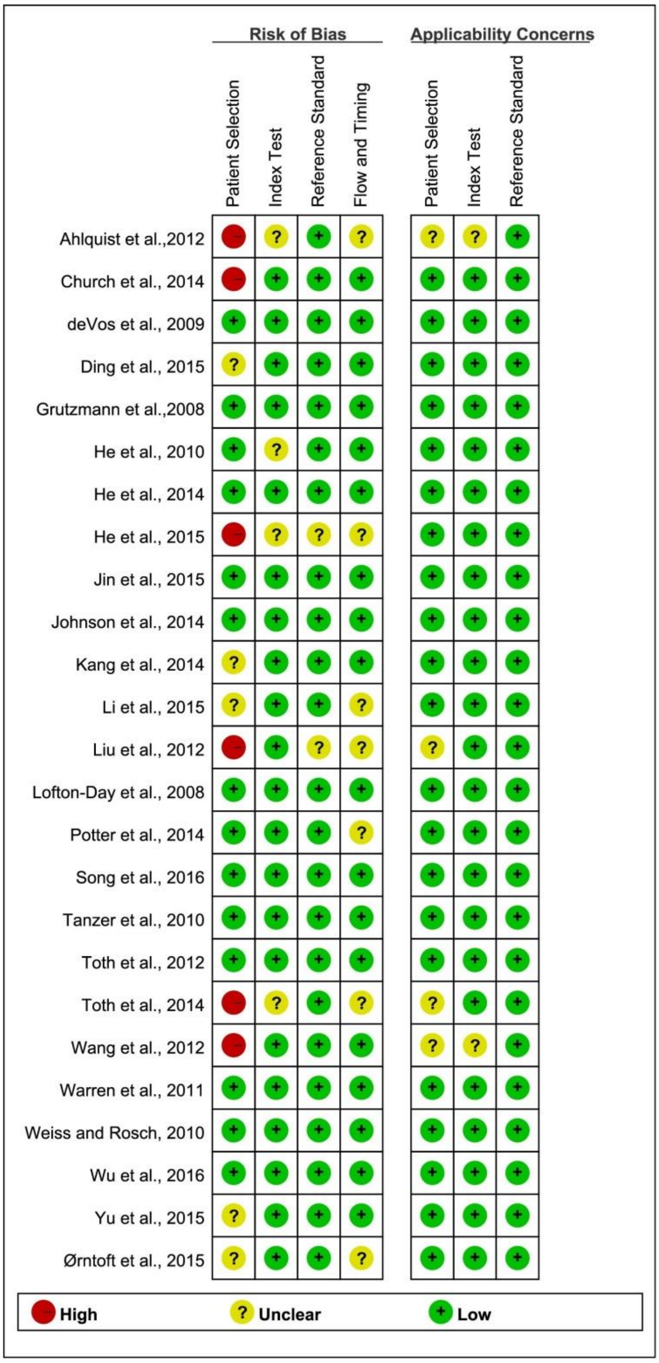

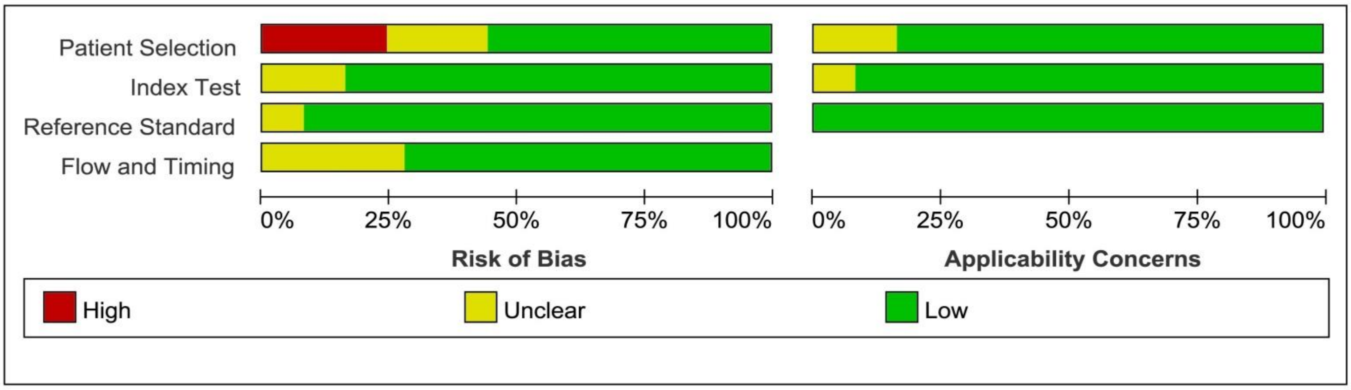

Supplementary Figure 1. Summary of quality of the included studies, according to the guidelines of quality assessment of diagnostic accuracy studies (QUADAS) criteria [8].

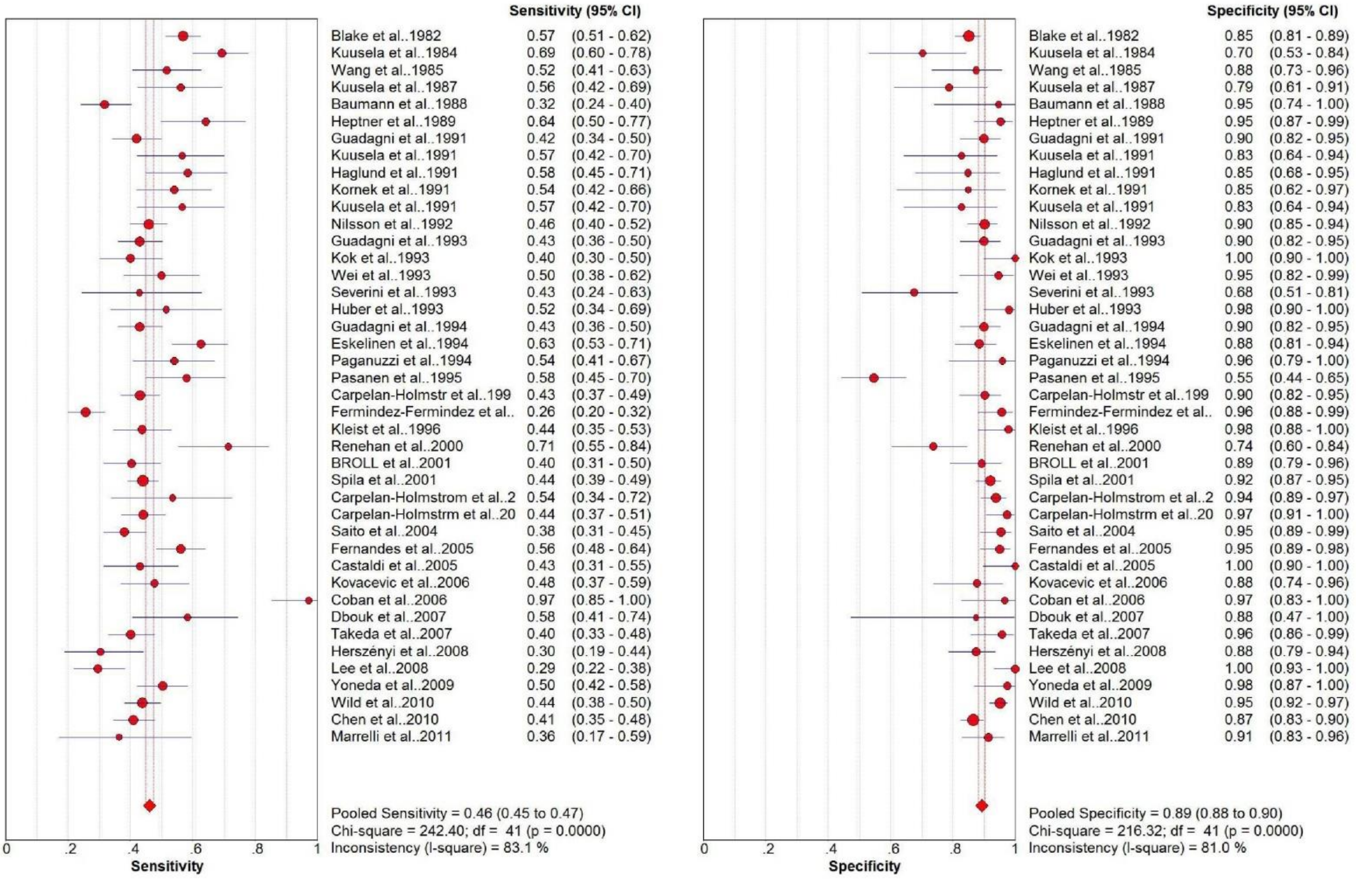

Supplementary Figure 2. Forest plot and estimated sensitivity and specificity for CRC detection with CEA with 95% confidence interval.

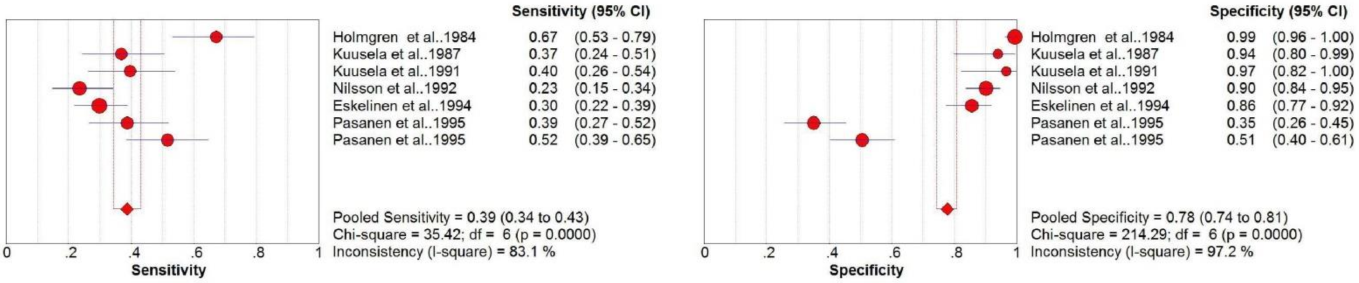

Supplementary Figure 3. Forest plot and estimated sensitivity and specificity for CRC detection with CA50 with 95% confidence interval.

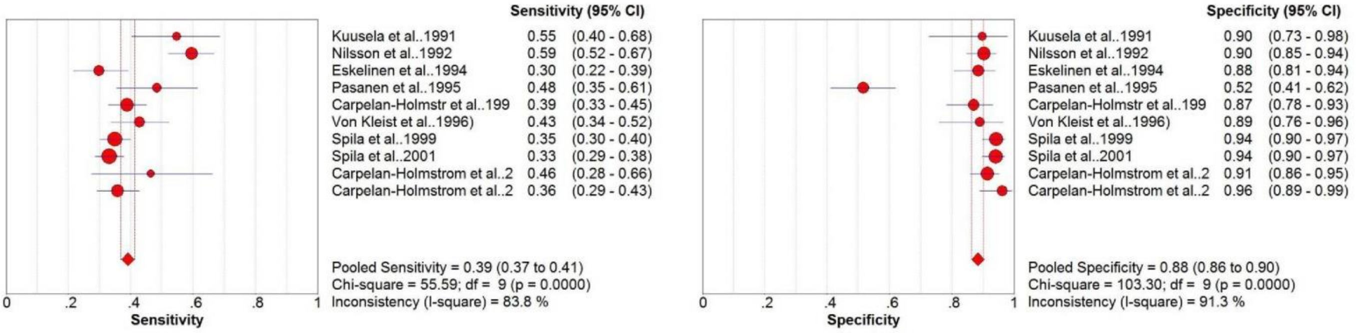

Supplementary Figure 4. Forest plot and estimated sensitivity and specificity for CRC detection with CA242 with 95% confidence interval.

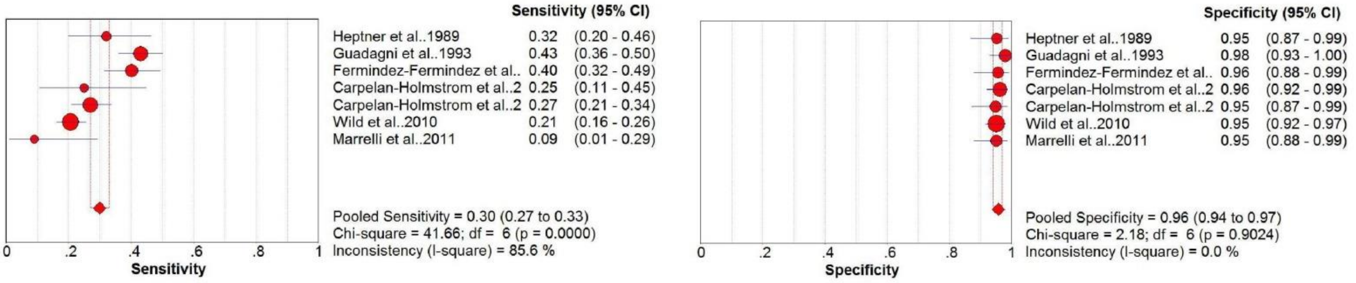

Supplementary Figure 5. Forest plot and estimated sensitivity and specificity for CRC detection with CA724 with 95% confidence interval.

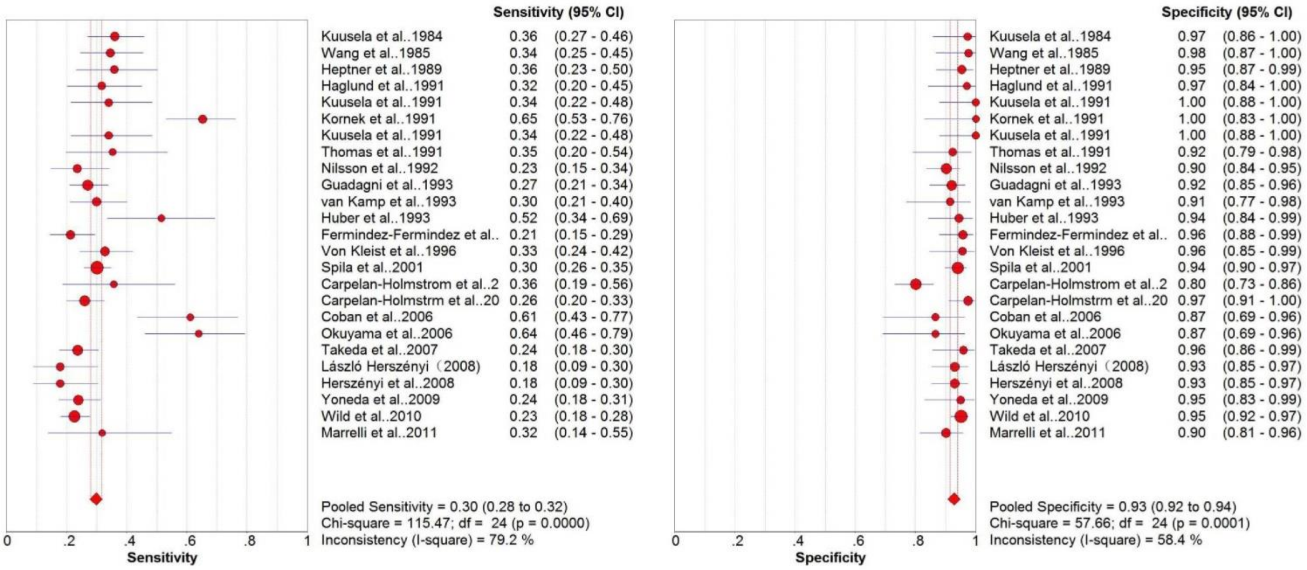

Supplementary Figure 6. Forest plot and estimated sensitivity and specificity for CRC detection with CA199 with 95% confidence interval.
